# Caveolin-1, a stress-related oncotarget, in drug resistance

**DOI:** 10.18632/oncotarget.5789

**Published:** 2015-09-27

**Authors:** Zhiyu Wang, Neng Wang, Pengxi Liu, Fu Peng, Hailin Tang, Qianjun Chen, Rui Xu, Yan Dai, Yi Lin, Xiaoming Xie, Cheng Peng, Honglin Situ

**Affiliations:** ^1^ Department of Mammary Disease, Guangdong Provincial Hospital of Chinese Medicine, The Second Clinical Collage of Guangzhou University of Chinese Medicine, Guangzhou, China; ^2^ Department of Breast Oncology, Sun Yat-sen Univeristy Cancer Center, State Key Laboratory of Oncology in South China, Collaborative Innovation Center for Cancer Medicine, Guangzhou, Guangdong, China; ^3^ Pharmacy College, State Key Laboratory Breeding Base of Systematic Research, Development and Utilization of Chinese Medicine Resources, Chengdu University of Traditional Chinese Medicine, Guangzhou, China

**Keywords:** Cav-1, cancer drug resistance, aerobic glycolysis, cancer stem cells, ABC transporters

## Abstract

Caveolin-1 (Cav-1) is both a tumor suppressor and an oncoprotein. Cav-1 overexpression was frequently confirmed in advanced cancer stages and positively associated with ABC transporters, cancer stem cell populations, aerobic glycolysis activity and autophagy. Cav-1 was tied to various stresses including radiotherapy, fluid shear and oxidative stresses and ultraviolet exposure, and interacted with stress signals such as AMP-activated protein kinase. Finally, a Cav-1 fluctuation model during cancer development is provided and Cav-1 is suggested to be a stress signal and cytoprotective. Loss of Cav-1 may increase susceptibility to oncogenic events. However, research to explore the underlying molecular network between Cav-1 and stress signals is warranted.

## INTRODUCTION

Multidrug resistance (MDR) can influence cancer clinical outcomes and ∼90% of cancer mortality is correlated to primary or acquired MDR and consequent metastases [1]. Thus, elucidating underlying molecular mechanisms of MDR can improve clinical outcomes for cancer patients. Currently three major mechanisms of MDR have been proposed: decreased chemotherapeutic uptake, absorption and distribution; aberrant drug metabolism that diminishes therapeutic efficacy, such as target alterations, DNA damage repair and cell death signaling pathway dysregulation; and finally, increased energy-dependent efflux of hydrophobic drugs by ATP-binding cassette (ABC) transporters [2].

Of these mechanisms, drug efflux by ABC transporters may explain more MDR. ABC transporters are encoded by 48 genes and classified into seven subfamilies (ABCA-ABCG) based on sequence homology and domain organization [3]. To date, sixteen members have been identified, but only three major ABC drug transporters, including ABCB1 (P-glycoprotein), ABCC1 (multidrug resistance protein 1, MRP1) and ABCG2 (BCRP), are thought to affect cancer chemosensitivity [4]. Numerous natural or synthetic inhibitors targeting ABC transporters were developed to reverse MDR. However, neither strong evidence nor clinical trial data are available to confirm the feasibility of these inhibitors for improving chemotherapeutic bioavailability or restoring drug sensitivity in resistant cancer patients [5]. Insufficient specificity, potency and intrinsic toxicity obstruct identifying ideal inhibitors to suppress ABC transporter activity. Also, substantial overlapping specificities exist among major ABC transporters, and some ABC proteins are expressed on vital organs for protective functions [6]. Thus, finding novel membrane transporters related to MDR is required for designing better drugs.

Caveolin-1(Cav-1) is an essential constituent protein of specialized membrane invaginations, referred to as caveolae. Research indicates that Cav-1 is a molecular hub, integrating transduction of multiple signals including Src, EGFR, HER2, and the mitogen-activated protein kinase (MAPK) cascade [7]. Cav-1 silencing was reported to result in activation of these survival signals and promote cancer transformation and initiation [7]. Meanwhile, loss of Cav-1 was frequently observed in various types of malignancies such as breast and colon cancers and ovarian carcinomas [8]. Therefore, Cav-1 may be a tumor suppressor gene. Recent work also indicates that Cav-1 may be a stress-related molecule mediating cancer drug resistance and metastasis. Cav-1 was up-regulated in MDR colon cancer cells, adriamycin-resistant breast cancer cells and taxol- and gemcitabine-resistant lung cancer cells [9]. Research indicated that Cav-1 expression was positively correlated to ABCB1 in various cancers such as acute myeloid leukemia [10-12]. Clinical findings also suggested that cancer patients with high Cav-1 expression had a worse chemotherapeutic response and worse progression-free survival or overall survival [13]. Moreover, Cav-1 was highly expressed on cancer stem cells (CSCs) and affected CSCs’ chemosensitivity [14]. Also, Cav-1 was found to be a critical regulator of aerobic glycolysis, a key metabolic switch influencing cancer chemosensitivity [15]. Finally, Cav-1 inhibition was shown to be correlated to autophagic induction, an important regulatory mechanism to protect cells from stress. However, autophagy overactivation can cause type II programmed cell death [16]. These data suggest that Cav-1 might be a stress-response protein and involved in the modulation of cancer chemosensitivity. Thus, targeting Cav-1 may offer a novel strategy for preventing cancer drug resistance and improving clinical outcomes.

Here, we summarize current evidence of a relationship between Cav-1 and cancer drug resistance, discuss pathophysiological implications of this pathway and propose a targeted therapeutic strategy. A greater understanding of Cav-1 and its role in modulating drug resistance will help us understand cancer therapy and discover better chemotherapeutics.

## CAV-1 SIGNALING

**Figure 1:**
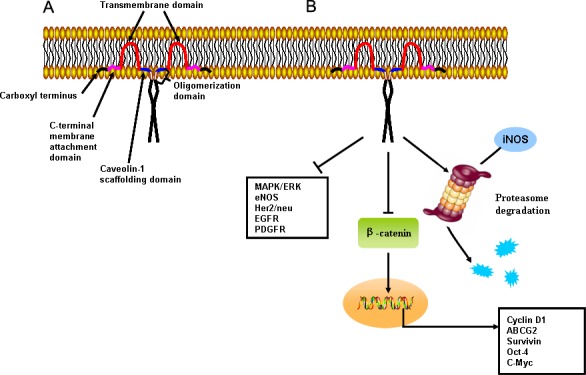
Primary structure and cellular signaling of Cav-1 **A.** Topology of membranous Cav-1. Cav-1 has a C-terminal spanning, transmembrane, N-terminal scaffolding, and oligomerization domains. Both C- and N-termini face the cytoplasm. **B.** The Cav-1 scaffolding domain interacts with and inhibits activity of well-known signaling regulators including G-protein coupled and tyrosine-kinase receptors, and eNOS and mitogen-activated protein kinases. In addition, the scaffolding domain may mediate proteasome degradation of iNOS and its inhibitory effects on transcriptional activity of β-catenin were noted.

Caveolins are 21-24 KDa membrane-associated proteins highly enriched in caveolae [17]. Each caveolae contains approximately 100 to 200 caveolin molecules formed by three principle members, Cav-1, -2, and -3 [18]. Cav-1 and -2 are ubiquitously co-expressed on epithelial and endothelial cells, fibroblasts, smooth muscle cells, adipocytes and pneumocytes, while Cav-3 expression is mainly restricted to muscle and glia cells [19]. The *CAV1* gene consists of three exons and maps to 7q31.1, where is close to a known fragile site (FRA7G) frequently deleted in cancer. *CAV1* encodes a 21- to 24-kDa integral membrane protein, and the topology of Cav-1 can be divided into three domains including a C-terminal spanning domain (residues 135-150), a transmembrane domain (residues 102-134), an N-terminal scaffolding domain (residues 82-101) and an oligomerization domain (residues 61-101). Both the C- and N-termini face the cytoplasm (Figure 1) [20]. Notably, the Cav-1 scaffolding domain (CSD) is a region that can mediate protein-protein interactions, such as Src-family tyrosine kinases, H-Ras, HER2, estrogen receptor, MAPK and G protein-coupled receptors (Figure 1) [7, 19]. Moreover, due to alternative splicing or initiation, Cav-1 exists in two isoforms, α or β. Cav-1β is distinct in that it has a 31 amino acid residue deletion at the amino terminus [7].

Cav-1 was initially described as a prevalent target for tyrosine phosphorylation in Rous sarcoma virus transformed chicken fibroblasts. Upon stimulation by agonists including insulin, epidermal growth factor (EGF), platelet-derived growth factor (PDGF), mechanical stress or oxidative stress, tyrosine 14 site of Cav-1 can be phosphorylated, subsequently transmitting extracellular signals via intracellular pathways [21]. Co-localization and co-fractionation bioassays demonstrated that Cav-1 not only interacted with many signal transduction proteins, such as Ras-p44/42 MAPK, Her-2, src family kinases and eNOS, but also inhibited their catalytic activity [22]. Many components of the Ras-p44/42 MAPK cascade localize within caveolae, including EGFR, PDGFR, H-Ras, Raf kinase, ERK1/2 kinases, Shc and Grb [23-25]. Suppression of Ras-p42/44 MAPK activity by treatment of PD98059 (a MAPK kinase inhibitor) was found to upregulate Cav-1 expression in Ras-transformed cells [26]. In contrast, Cav-1 overexpression inhibited Ras-p42/44 MAPK signaling by acting as an endogenous inhibitor of EGFR, MEK-1 and ERK1/2 *in vitro* and *in vivo via* the CSD domain [27]. Similar reciprocal regulation was also observed between Cav-1 and c-ErbB2, a proto-oncogene encoding Her-2 in human breast carcinomas. Cav-1 expression is significantly reduced in mammary tumors of c-ErbB2 transgenic mice [28]. Conversely, Cav-1 upregulation blocks Her-2 mediated signal transduction *in vivo* by directly inhibiting the Her-2 autophosphorylation *via* its CSD domain [27]. In addition, Cav-1 is known to bind and sequester Src family kinases in an inactive configuration [29, 30]. Accordingly, Cav-1 deprivation leads to a marked increase in Src family tyrosine kinase activity, and therefore significantly contributes to the migration and anchorage-dependent growth of osteosarcoma cells [31]. Meanwhile, Cav-1 loss is suggested to trigger the phosphatidylinositol 3-kinase (PI3K)/Akt pathway and induce cell transformation in mammary epithelial cell line MCF-10ACE [32]. Furthermore, Cav-1 was shown to participate in the formation of a multi-protein complex, which includes E-cadherin/β-catenin and helps sequester β-catenin to the membrane, thereby precluding β-catenin/Tcf-Lef-dependent transcription of genes such as survivin, COX-2 and cyclin D1 [33]. Notably, Cav-1 was also shown to inhibit eNOS enzymatic activity and nitric oxide production in a Ca^2+^/calmodulin-dependent fashion [34]. Overall, Cav-1 was reported to have a central role in regulating cell survival, proliferation, and apoptosis. Elucidation of Cav-1 in cancer development and progression may be significant for improving patient prognosis and preventing tumor onset.

## CAV-1 AND CANCER DEVELOPMENT

Despite advancements in understanding Cav-1 structure and function, the relationship between dysfunctional Cav-1 and tumorigenesis is unclear. Downregulation of Cav-1 and its tumor suppression function has been validated in breast, colon, and ovarian cancer and soft-tissue sarcomas. First, studies show that Cav-1 is negatively associated with cancer cells’ transformation. Xie's group found that Cav-1 expression was significantly down-regulated in 3-phosphoinositide-dependent protein kinase-1 (PDK1)-mediated transformation of mammary epithelial cells [35]. Another study revealed that stable expression of HPV E6 viral oncoprotein in NIH 3T3 cells increased Cav-1 protein. In contrast, adenoviral-mediated overexpression of Cav-1 in SiHa cells, a human cervical squamous carcinoma cell line, abrogated their anchorage-independent growth in soft agar [36]. Similar findings were also validated in alveolar rhabdomyosarcomas. The Cav-1 expression was found either undetectable or very low in alveolar rhabdomyosarcomas cell lines and tumor samples, whereas Cav-1 reintroduction impaired cells clonogenic capacity and promoted features of muscular differentiation [37]. Next, Cav-1 associates with known tumor suppressors. In MCF-7 cells, transfection of murine Cav-1 cDNA led to increased BRCA1 transcription and protein overexpression in a P53 dependent manner [38]. BRCA1 was also found to activate the Cav-1 promoter region and induce redistribution of Cav-1 from the cytoplasm to the cell membrane [39]. Several studies indicate that Cav-1 expression was positively correlated with PTEN in mammalian cells, and Cav-1 restoration in fibroblasts could elevate membrane PTEN, inhibit Akt phosphorylation, and suppress proliferation [40]. In addition, a series of tumor suppressors including deleted in liver cancer 1 (DLC-1), INK4a and RB, were revealed to be closely correlated to Cav-1 status [41-43]. Finally, *in vivo* studies confirmed that the absence of Cav-1 resulted in mammary ductal hyperplasia, precocious lobuloalveolar development and gestational lactation [44]. Meanwhile, hybridization of Cav-1 knockout mice with tumor prone transgenic mice (MMTV-PyVT) decreased tumor development latency, doubled tumor burden and lung metastatic lesions [28, 45]. Accumulating evidence indicates that Cav-1 was significantly reduced or absent in mammary tumors from MMTV-c-Myc, -Her2, -Src, -Ha-Ras and p53 null transgenic mice [46]. Notably, clinical studies with microarray analysis and immunohistochemistry revealed that Cav-1 expression was equally decreased in either mammary invasive lobular carcinomas or invasive ductal carcinomas [47]. Similar findings were also confirmed in other malignancies including colon and ovarian cancers and osteosarcomas [47].

**Figure 2:**
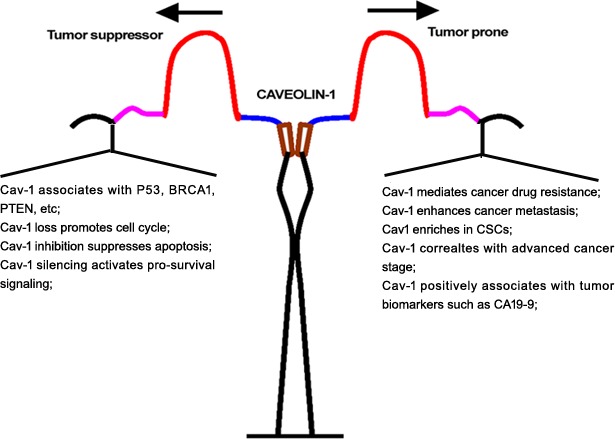
Duplex role of Cav-1 in cancer origination and metastasis

Intriguingly, opposing evidence supports a role for Cav-1 as a tumor-promoting protein. Cav-1 in pancreatic ductal adenocarcinoma is positively associated with tumor size, grade and stage and Cav-1 expression is correlated to Ki67, P53 in tumor tissues and serum CA 19-9 serum [48]. Other work suggested that Cav-1 expression was significantly correlated to tumor diameter, histological grade and poor prognosis in pancreatic cancer, and indicated that Cav-1 might be an independent negative predictor of survival [49]. Similar findings were confirmed in renal cancer. Campbell's group suggested that high Cav-1 expression was closely correlated to larger and higher grade tumors, as well as microvessel density, and survival analysis confirmed that increased Cav-1 staining portended shorter survival [50]. In hepatocellular carcinoma, Cav-1 expression was also markedly upregulated in specimens or cell lines, and Cav-1 upregulation positively correlated with histological differentiation, venous invasion, intrahepatic metastasis and VEGF expression [51]. Even in breast cancer, a strong association was found between elevated Cav-1 and a basal-like phenotype [52]. Cav-1 expression was upregulated in inflammatory breast cancer cells and tissues [53] and Cav-1 overexpression significantly contributed to cancer metastasis. With using proteomic identification, Cav-1 was found to be elevated in metastatic hepatocellular cancer cell lines [54]. A microarray analysis also supported Cav-1 as a metastasis-related gene by comparing gene expression profiles between weakly and highly invasive breast cancer cells [55]. Specifically, Cav-1 silencing was effective for reducing metastatic activity of prostate cancer cells *in vitro* and *in vivo*, whereas Cav-1 restoration was shown to enhance the motility of tumor cells in lung cancer, hepatocellular carcinoma and melanoma [56]. In addition, as early as 2001, Hayashi's group reported that the Cav-1 mutant form P132L was identified in 16% of primary breast cancers and mutation-positive cases were mostly invasive scirrhous carcinomas [57]. Subsequent studies revealed that Cav-1 (P132L) behaves in a dominant-negative manner, causing mislocalization and intracellular retention of WT Cav-1 [58]. Mechanistic investigation further verified that Cav-1 (P132L) upregulated the expression of estrogen receptor-α and significantly increased cell migration, invasion and experimental metastasis. Gene profile analysis demonstrated that Cav-1 (P132L) mutation was associated with stem cell/metastasis-associated gene signature, including Cdcp1, Cyr61, Fox1a, Krt14, Pif, Tacstd1, Tnc and Wnt10a [59]. However, several studies also claimed that the P132L mutation was not identified in breast cancer samples, either by direct DNA sequencing or restriction fragment length polymorphism analysis [60-63]. Thus, reevaluating the existence and frequency of P132L mutations in breast cancer is needed. Here, we summarize conflicting evidence regarding Cav-1 during cancer development (Figure 2). Cav-1 may be a duplex signal during cancer progression, and its expression status might be closely correlated to tumor stage. During cancer transformation and initiation, Cav-1 acted as a tumor suppressor gene in breast, lung, cervical, gastric, glioma and pancreatic malignancies [64-69]. Cav-1 loss not only inactivate tumor suppressors BRCA1, P53 and PTEN but also promoted cell cycle progression and activated pro-survival signaling such as PI3K/Akt and MAPK [36, 38, 70]. However, with cancer advanced progression, Cav-1 re-expression was observed in various cancers including breast, lung, prostate, liver, ovarian, pancreas, melanoma, thyroid, colorectal, gastric, renal and pleomorphic malignancies [51, 54, 57, 71-79]. Cav-1 overexpression was found in metastatic lesions of breast cancer and was closely correlated with tumor stage and clinical prognosis [80]. Meanwhile, Cav-1 positively correlated with CSC self-renewal and the tumor biomarker CA19-9 in cancer patients [81, 82]. These data suggest that validating Cav-1 in cancer development with time point-guided detection and genetic intervention tools to investigate the function of Cav-1 in different cancer stages are needed. Although the underlying mechanisms of Cav-1 fluctuation during cancer progression are unclear, studies to better understand the therapeutic and prognostic values of Cav-1 for cancer—especially for chemosensitivity and chemoresistance—are underway.

## IMPLICATION OF CAV-1 IN CANCER DRUG RESISTANCE

Studies suggest that Cav-1 is a chemotherapy-response gene independent of cancer type. Cav-1 expression was significantly upregulated in a series of drug-resistant cancer cells. In a taxol-resistant (9-fold resistant to taxol) lung cancer cell line A549, Cav-1 expression increased 3.4-folds compared to the parental cell line. Increasing resistance taxol to 17-fold, increased Cav-1 expression 9.5-fold [83]. Meanwhile, increased Cav-1 expression has been reported in other drug-resistant cancer cells, such as SKVLB1 cells resistant to vinblastine, HT-29 cells resistant to colchicine and MCF-7 cells resistant to adriamycin [83, 84]. In addition, Cav-1 expression is significantly induced after treating lung cancer cells with etoposide or bleomycin, indicating that Cav-1 might be a stress-related protein [11]. Thus, genetic and pharmacological studies confirmed a correlation between Cav-1 and drug resistance. Cav-1 silencing increased chemosensitivity or radiosensitivity in diverse cancer cells such as Ewing's sarcoma cells A4573, Madin-Darby canine kidney cells MDCKII and the pancreatic cancer cell line MiaPaCa2 [84-86]. Cav-1 silencing increased susceptibility of human renal carcinoma cells to doxorubicin-induced apoptosis and inhibited lung metastasis. In contrast, Cav-1 restoration promoted resistance to chemotherapy-induced apoptosis in Ewing's sarcoma cells [87]. Cav-1 knockout mice had less survival compared with the Cav-1 wide-type mice after whole body irradiation [88]. In a prostate cancer animal model, combined dasatinib and anti-Cav1 antibody treatment or sunitinib and anti-Cav1 antibody produced greater tumor regression than either treatment alone [89]. More importantly, a clinical study revealed that Cav-1 expression was correlated to chemotherapy response in non-small lung cancer. Patients who had tumors samples with strong Cav-1 staining had less response to therapy and worse progression-free and overall survival [90]. Similar findings were also confirmed on gastric cancer. Cav-1 was positively associated with tumor stage and nodal status, and was inversely correlated with the response to ECF (epirubicin, cisplatin and 5-fluorouracil) chemotherapy administrated to gastric cancer patients [91]. Another study validated the value of Cav-1 in predicting chemoresponse. A comprehensive methylation microarray analysis of 49 patients with advanced non-small cell lung cancer (NSCLC), suggested that Cav-1 was a powerful predictor for therapeutic response after platinum-taxane based treatment [92]. Cav-1 methylation was significantly associated with improved overall survival [92]. Therefore, the underlying mechanisms between Cav-1 and cancer chemosensitivity modulation warrant study.

## CAV-1 AND ABC TRANSPORTERS

Since the discovery of the first ABC family member, ABCB1, in multidrug resistant ovarian cancer cells in 1976, research to identify drug transporters contributing to cancer drug resistance has been underway. Currently, at least seven ABC subfamilies (A-G) encoded by 48 genes have been identified [4]. Among them, ABCB1 is the most well-studied target to regulate cancer drug resistance and develop chemosensitizing agents. Numerous studies show that ABCB1 can extrude hydrophobic compounds central to most chemotherapeutic regimens, including vinca alkaloids, anthracyclines, taxanes and epipodophyllotoxins [93]. In addition, some tyrosine kinase inhibitors such as imatinib, nilotinib and erlotinib, can be exported by ABCB1 [94]. ABCB1 is normally expressed in the transport epithelium of the liver, kidney and gastrointestinal tract so it is difficult to justify the role of ABCB1 for predicting chemosensitivity of tumors originating from these sites. However, clinical studies revealed a positive correlation between ABCB1 expression and poor prognosis in breast cancer, sarcoma and certain types of leukemia, because ABCB1-positive biopsies from these cancers can be compared with ABCB1-negative patients [95]. Interestingly, a close association has been observed between Cav-1 and ABCB1. Both proteins are predominantly located in the Lubrol-based detergent-insoluble glycosphingolipid-enriched membrane domains and co-immunoprecipitation assays confirm that Cav-1 interacts with ABCB1 in brain capillaries, rat brain endothelial, Chinese hamster ovary and breast cancer cells [96]. Sucrose gradient fractionation revealed that Cav-1 co-localized with ABCB1 in lipid rafts. The interaction seems to be mediated by a Cav-1-binding motif in the N-terminal portion of ABCB1 (37-FSMFRYSNW-45) [97]. Clinical studies indicated a positive correlation between Cav-1 and ABCB1 mRNA in acute myeloid leukemia independent of tumor stage [9]. However, studies suggested that Cav-1-regulated chemosensitivity was ABCB1 independent. In ABCB1 deficient taxol-resistant A549 cancer cells, Cav-1 upregulation was observed [83] and Cav-1 overexpression correlated with little ABCB1 protein expression in human ovarian cancer cells, and ABCB1 overexpression was not localized in caveolae [98]. Although accumulating evidence suggested a potential molecular interaction between Cav-1 and ABCB1, detailed signaling pathways between the two molecules are unknown, and more studies of Cav-1 to understand the transcriptional and translational expression of ABCB1 are urgently needed.

Other than ABCB1, ABCG2 is a high-capacity transporter widely expressed in drug resistant cancer cells. ABCG2 substrates include organic anion conjugates, nucleoside analogs, tyrosine kinase inhibitors, organic dyes, cytotoxic drugs, methotrexate and food toxins or carcinogens. Compared with ABCB1, ABCG2 is a mediator of MDR in breast, colon, small cell lung, ovarian, gastric and intestinal cancers and melanomas [99]. Several reports also indicated a potential correlation between Cav-1 and ABCG2. Cav-1 silencing in MDCKII-BCRP cells led to a reduction of ABCG2 activity by 35% [86]. Co-immunoprecipitation experiments demonstrated a physical interaction between Cav-1 and ABCG2, which were co-localized in detergent-resistant membranes [11]. Another study indicated that Cav-1 was highly expressed on breast cancer stem cells or side-population cells, which were enriched with ABCG2. Furthermore, Cav-1 silencing may decrease ABCG2 expression *via* accelerating β-catenin proteasomal degradation [14]. Because CSCs are considered a root of drug resistance, the study also suggested a role for Cav-1 in regulating chemosensitivity mediated by stem cells.

## CAV-1 AND CSCS

The first compelling evidence of CSCs came from acute myeloid leukemia in 1997 which indicated that only CD34^+^CD38^−^ cells could passage hematopoietic malignancy in NOD/SCID mice after serial dilution [100]. Similar phenomena were subsequently validated in various types of cancers, such as breast cancer, hepatocellular carcinoma, gastric and lung cancers. Based on accumulating evidence, the American Association for Cancer Research (AACR) defined CSCs as “a cell within a tumor that possesses the capacity to self-renew and to cause heterogeneous lineages of cancer cells that comprise the tumor” [101]. Therefore, self-renewal and multi-differentiation abilities are considered basic characteristics of CSCs. Research with CSCs suggests that they are relatively resistant to chemotherapy and radiotherapy. High expressions of ABCG2, ABCB1, or ABCC1 were validated in CSCs in various cancers, and the ability of CSCs to extrude Hochest 33342 by ABCG2 was used to develop the “side population” method, which is frequently used to isolate stem-like cells from primary tumors or cancer cell lines [102]. Clinical studies also revealed a positive correlation between CSCs population size and poor prognosis in cancer patients. Neumeister's group applied CD44 and aldehyde dehydrogenase 1 (ALDH1) biomarkers to define CSCs in formalin-fixed paraffin-embedded breast cancer tissue. Through a retrospective analysis of 642 patients, they found that specimens with high expression of both markers predicted worse outcomes (*P* = 0.0003), and multivariate analysis confirmed that the marker combination is independent of tumor size, histological grade, nodal status, ER, PR, and HER2 status [103]. Meanwhile, another study reported that during neoadjuvant chemotherapy of breast cancer patients, ALDH1-positive tumors were significantly associated with a low pathological complete response. More importantly, the proportion of ALDH1-positive cancer cells increased dramatically (*P* < 0.001) after neoadjuvant chemotherapy, indicating that CSCs were relatively chemoresistant [104]. Similar to breast cancer, a meta-analysis of hepatocellular carcinoma patients revealed that the presence of CSCs was significantly associated with a poor histological grade (OR = 3.16, *P* = 0.003), overall survival (HR = 1.62, *P* < 0.00001) and disease-free survival (HR = 1.85, *P* < 0.00001). Notably, there were no significant correlation between the populations of CSCS and tumor size, tumor stage, hepatitis and cirrhosis, indicating that CSCs subset might be an independent factor influencing the clinical outcomes of cancer patients [105]. At present, mechanisms underlying therapeutic resistance of CSCs have not been fully elucidated. However, several mechanisms have been suggested, including: (i) stem cells remain quiescent, making them resistant to cell-cycle active drugs; (ii) high DNA repair ability; (iii) high expression of drug efflux proteins such as ABCG2; and (iv) most current therapies do not target signaling pathways regulating CSCs self-renewal. Elucidation of molecular networks regulating drug sensitivity of CSCs might be essential for eliminating cancer cells.

In recent years, increasing interests have been paid to explore the possible link between Cav-1 and stem cells. It was found that in human mesenchymal stem cells (MSCs), Cav-1 expression was significantly increased when cells were induced to senescence, while Cav-1 lost remarkably increased the proliferation of MSCs, indicating that Cav-1 might be closely associated with the maintenance and self-renewal abilities of stem cells [106]. Cav-1 has positive effects on mediating estradiol-17β 0or fibronectin-induced mouse embryonic stem cell proliferation and DNA synthesis via RhoA-PI3K/Akt-ERK1/2 pathway [107]. With regard to the multi-differentiation ability of stem cells, several reports also indicated that Cav-1 knockdown could enhance human MSC osteogenesis, and bone marrow MSCs from the Cav-1 knockout mice had greater osteogenic potential [106]. Cav-1 null mouse mammary gland also showed dramatically accelerated lobuloalveolar development, early milk production and a premature lactation phenotype during lactation, indicating that Cav-1 lost might favor the differentiation process of mammary epithelial cells [108]. Mechanistic studies suggest that Cav-1 expression and caveolar structure help maintain pluripotency marker expression (Oct4, Sox2, FoxD3, Rex1) in mouse embryonic stem cells [109]. All of these findings suggested that Cav-1 may influence self-renewal and multi-differentiation of stem cells. With regard to CSCs, it was found that nitric oxide administration promoted CSC-like phenotype *via* elevating Cav-1 expression in lung cancer cells [110]. Also, through genome-wide transcription profile analysis, Cav-1 was identified as a key protein for determining the formation of lung CSCs in a carbon nantotube-induced carcinogenesis animal model. Cav-1 was confirmed to be enriched in lung CSCs and significantly promoted tumorigenesis and metastasis through P53 dysregulation [81]. Emerging studies suggested that Cav-1 may regulate chemoresistance of CSCs. Yuan's group found that epirubicin increased the activity of the human Wnt6 promoter through Cav1-dependent binding of β-catenin to the proximal Wnt6 promoter in gastric cancer, which is considered an important regulator of CSCs [91]. Another study of Wang's group confirmed that Cav-1 was upregulated in breast CSCs compared with non-CSCs. In addition, Cav-1 silencing sensitized breast CSCs by limiting their self-renewal ability but promoting differentiation through β-catenin regulation. Meanwhile, *in vivo* animal models indicated that Cav-1 silencing significantly impaired tumorigenicity and chemoresistance of breast CSCs [14]. Thus, current research for Cav-1 in mediating therapeutic resistance of CSCs is limited to a few cancer types, and a more comprehensive approach is needed to validate a critical role of Cav-1 for determining the therapeutic response of CSCs in cancers, and to clarify the physiological function of Cav-1 in maintaining stem cell survival and its molecular network. The hybridization of Cav-1-null mice with a spontaneous cancer model may be helpful.

## CAV-1 AND WARBURG EFFECT

Cancer cells can be distinguished from normal cells by several hallmarks; one is that cancer cells have a fundamentally different bioenergetic metabolism from non-neoplastic cells. In normal cells, energetic metabolism mostly relies upon mitochondrial oxidative phosphorylation, which consumes glucose that is finally metabolized to carbon dioxide, water and ATP. In contrast, cancer cells have developed altered metabolism that permits greater proliferation rates. Cancer cells could predominantly produce energy by glycolysis followed by lactic acid fermentation, even in the presence of oxygen—known as the “Warburg Effect” [111]. Currently, the phenomenon has been exploited clinically to detect tumors by fluorodeoxyglucose positron emission tomography (FDG-PET). Some key enzymes involved in the glycolysis pathway were also developed as promising targets for cancer therapy and drug development. LDH-A is one such attractive target in the glycolytic pathway because its expression is largely confined to skeletal muscle. Moreover, human subjects with LDH-A deficiency only experience myoglobinuria under intense anaerobic exercise, and individuals with a complete lack of the LDH-A subunit have been documented to have no apparent increase in hemolysis [112]. Numerous studies confirm that overexpression of LDH-A in various types of cancer, and LDH-A inhibition in cancer cells could result in significant stimulation of mitochondrial respiration, decreased mitochondrial membrane potential and finally lead to cancer cell death [113]. Interestingly, recent reports also revealed a close association between the Warburg effect and development of cancer drug resistance. Zhou's group studied a parental breast cancer cell line MDA-MB-435 and reported a significant elevation of LDH-A protein in taxol-resistant 435R1 and 435TRP cell lines. Meanwhile, taxol treatment led to a dose-dependent increase of LDH-A expression in MDA-MB-435 cells, and LDH-A stability appeared to be elevated after taxol administration, indicating that LDH-A might be biomarker for detecting chemosensitivity [114]. In addition, LDH-A silencing or the use of a glycolysis inhibitor oxamate significantly re-sensitized the taxol-resistant cells to taxol treatment. Notably, combination treatment with LDH-A siRNA and oxamate increased sensitivity in taxol-resistant cell viability compared with either agent alone [115]. Also, compared to gemcitabine-sensitive AsPC-1 cells, gemcitabine-resistant cells Panc-1 and Pan-1^GemR^ had more mRNA for many glycolytic enzymes including hexokinase II, glucose phosphate isomerase, 6-phosphofructo-2-kinase/fructose-2,6-biphosphatase 4, aldolase, pyruvate kinase M2, and pyruvate dehydrogenase kinase 3. Upon exposure to glucose transporter (GLUT) inhibitor CG-5, the proliferation of Panc-1 and Pan-1^GemR^ was greatly limited, and was accompanied with suppression of Akt-mTOR signaling, activation of amp-activated protein kinase (AMPK), and suppressed expression of a series of oncogenic proteins, such as cyclin D1, Sp1, and Skp2 [15]. Interestingly, glycolytic activity of tumor stromal cells was also reported to contribute to cancer drug resistance. Breast cancer MCF-7 cells are is preliminarily sensitive to tamoxifen. However, when co-culturing breast cancer cells MCF-7 with cancer-associated fibroblasts, MCF-7 cells gained resistance to tamoxifen. Mechanistic studies indicated that glycolytic fibroblasts produced lactate that was sufficient to provide nutrients to support cancer cell survival [116]. Thus, the Warburg effect is correlated to the development of chemoresistance and tumor progression, and elucidation of key modulators involved in the Warburg effect may offer strategies for treating cancer drug resistance.

Considering the significance of Cav-1 in nutrient delivery and cancer development, emerging studies are underscoring the role of Cav-1 in regulating cancer metabolism. Raikar's group reported that Cav-1 affects expression of glycolytic enzymes phosphofructokinase and aldolase and that co-localization of Cav-1 and phosphofructokinase or aldolase occurred throughout A7r5 cells. Overexpression of Cav-1 shifted phosphofructokinase and aldolase towards the plasma membrane, indicating that Cav-1 may influence activity and cellular distribution of glycolytic enzymes [117]. Also, Cav-1 loss significantly downregulated glucose uptake, intracellular ATP and lactate accumulation in colon cancer cells, whereas Cav-1 overexpression increased glucose uptake and ATP production by stimulating HMGA1-medaited transcription of the glucose transporter SLC2A3/GLUT3, which was reported to play a significant role in the development of drug resistance [118]. Similar findings were also observed in prostate cancer for which Cav-1 overexpression significantly elevated glucose uptake and lactate production, and this was accompanied with upregulation of glucose transporter 3 and hexokinase 2. However, these events did not alter enolase1, LDHA, pyruvate kinase M2, and pyruvate dehydrogenase kinase 1 protein. Mechanistic studies indicated that Cav-1-induced aerobic glycolysis was mediated through the Akt-mTORC1 pathway and independent of β-catenin signaling [119]. Recently, evidence suggests a function for Cav-1 in tumor stroma. Immunohistochemical studies of human breast cancer specimens revealed that biopsies lacking stroma Cav-1 overexpressed key glycolytic enzymes, such as PKM2 and LDH. Meanwhile, transcriptional profiles of Cav-1-negative (−) tumor stroma revealed up-regulation of 238 genes and downregulation of 232 genes compared to Cav-1 positive (+) breast cancer stroma. Notably, a significant gene upregulation of hypoxic target genes (65 transcripts), glycolysis/pyruvate metabolism (15 transcripts), and autophagy (22 transcripts) were involved in the Cav-1-negative (−) tumor stroma [120]. In a Cav-1 knockout mice model, bone-marrow-derived stroma cells upregulated both myofibroblast markers and glycolytic enzymes [121]. Accelerated glycolytic activity of stroma fibroblasts provided nutrients to support cancer cell survival and growth—there was high expression of monocarboxylate transporter 4 (MCT4, for lactate extrusion) in cancer associated fibroblasts, whereas adjacent breast cancer cells expressed MCT1 (for lactate uptake), suggesting metabolic coupling occurred between cancer cells and stromal fibroblasts [116]. Lactate secreted from fibroblasts could be reabsorbed by cancer cells to enter the tricarboxylic acid cycle, and be metabolized into ATP and substrates for DNA or protein synthesis. This hypothesis was validated by increased tumor growth observed after co-injecting breast cancer cells and CL4 fibroblasts into nude mice cancer xenograft [122]. In addition, several studies suggest that stromal Cav-1 loss is an independent factor associated with poor clinical prognosis of breast cancer patients, indicating that stromal Cav-1 loss may assist cancer metastasis or drug resistance [82, 123, 124]. Thus, the effects of Cav-1 on aerobic glycolysis may be opposite in cancer cells and tumor stroma. Combined targeted therapy should be considered when designing novel treatment strategies based on Cav-1 to overcome cancer metastasis or drug resistance. In addition to direct silencing of Cav-1 to target cancer cells, it is necessary to protect Cav-1 from downregulation in tumor stroma. Thus, it is more prudent to combine Cav-1-targeting agents with antibodies against tumor specific antigens, such as EGFR, HER2, and mucins, etc. On the other hand, combining Cav-1 silencing agents with glycolysis inhibitors to prevent glycolytic activation in tumor stroma may also avoid the risk of fueling cancer cell growth.

## CAV-1 AND AUTOPHAGY

Autophagy is emerging as a crucial response to metabolic and therapeutic stresses, attempting to maintain cellular homeostasis via eliminating excessive or unnecessary proteins and damaged organelles through lysosome degradation [125]. However, persistent stress can promote extensive autophagy, inducing type II programmed cell death. Increasing evidence suggests that cytotoxic anti-cancer agents including other drugs, tyrosine kinase inhibitors imatinib and cetuximab, tamoxifen and cyclooxygenase inhibitors may induce autophagy in cell culture and animal models [126]. Interestingly, autophagic inhibition opposed camptothecin-induced cytotoxicity in osteosarcoma, whereas autophagic induction by roscovitine assisted cell killing by doxorubicin, indicating that might become a novel strategy to enhance cancer chemosensitivity [127, 128]. Additionally, autophagic induction may overcome drug resistance during cancer therapy. The constitutive expression of ABCB1 in hepatocellular cancer cells was positively linked to Bcl2 and mTOR overexpression, rendering these cells resistant to autophagy [129]. In contrast, autophagic induction by vocamine activated autophagic cell death in doxorubicin-resistant osteosarcoma cells, which was accompanied by decreased ABCB1 expression [130]. Thus, autophagy is important for regulation of cancer drug resistance and molecules that can induce autophagic cell death may be potential targets for regulating chemosensitivity.

At present, genetic screens in yeast have identified a large family of core autophagy-related genes (ATG), such as Atg1, Atg4, LC3/Atg8, and BECN1 (Beclin-1) [131]. Moreover, mTOR also serves as the main regulator of autophagy. Under nutrient deprivation, hypoxia, genomic instability or other stress, mTOR suppression triggers the autophagic cascade and inhibits cell proliferation [132]. Mechanistic work revealed that mTORC1 activation can negatively regulate autophagy by phosphorylating a complex of autophagic proteins such the Unc51-like kinases (ULK1/2), which interfere with the formation of autophagosomes [133]. Furthermore, as a central sensor of cellular nutrient status or energy, AMPK is involved in autophagic regulation and may serve as an upstream regulator of mTORC1 [134]. AMPK activation can subsequently trigger TSC2 to repress mTORC1 and activate autophagy. In addition, research suggests that AMPK may directly phosphorylate multiple sites in ULK1 (S317, S467, S555, T575, S637, and S777) and promote ULK1 function in autophagy. Even with molecular elucidation of autophagic regulation, more detailed signaling networks await exploration.

Intriguingly, recent work indicates a potential correlation between Cav-1 and autophagy. Lay reported that in Cav-1 knockout adipocytes, autophagy marker LC3-II/LC3-I ratios were significantly elevated and this was validated in Cav-1 null mice using electron microscopy and LC3II/LC3-I ratios in adipose tissue [135]. Similar work confirmed that mammary pads of Cav-1 null mice generated a lethal tumor microenvironment and constitutively underwent autophagy by increased oxidative stress and inflammatory cytokines, which provides recycled nutrients to feed cancer cells [136]. Shi's group also demonstrated that Cav-1 deficiency could induce autophagy via enhancing lysosomal function and autophagosome-lysosome fusion in human breast cancer cells [16]. A recent mechanistic study confirmed that Cav-1 can directly interact with and regulate expression of ATG12-ATG5, an ubiquitin-like conjugation system crucial for autophagosome formation, in lung epithelial Beas-2B cells [137]. Cav-1 deletion increased basal and starvation-induced ATG12-ATG5 and autophagy, whereas mutation of the Cav-1 binding motif on ATG12 disrupted this interaction and augmented autophagy [138]. Thus, Cav-1 loss may cause autophagic flux. Current evidence also explains why Cav-1 expression is restored in metastatic or drug-resistant cancer cells. Thus, Cav-1 re-expression can cause autophagy suppression, which can inhibit lysosomal degradation of ABC transporters, but also block autophagic cell death. Finally Cav-1 enriched cancer cells acquired drug-resistant properties and stem-like characteristics [9, 14]. Although indirect evidence indicates that Cav-1 is positively associated with expression of ABC transporters and a population of stem cells, the role of autophagy in mediating these molecular events requires verification. Meanwhile, autophagic activation by Cav-1 silencing may be a novel strategy for overcoming cancer drug resistance.

## PERSPECTIVE

**Figure 3:**
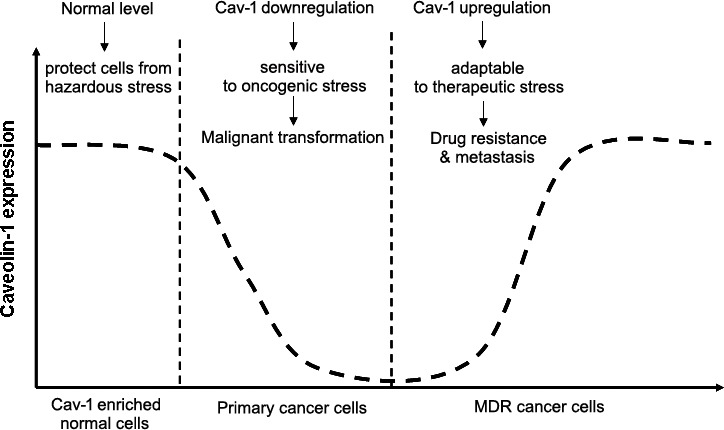
Cav-1 may act as a stress-response signaling during cancer development in Cav-1 enriched cells When Cav-1 is maintained at the normalized level, it protects Cav-1 enriched normal cells from hazardous damage. However, when Cav-1 declines below the threshold, cells will become sensitive to oncogenic stress and initiate malignant transformation. With cancer progression to the advanced stage or is treated with cytotoxic agents, Cav-1 may re-overexpress to help cancer cells escape death, causing drug resistance and metastasis.

After drug treatment, cancer cells must activate self-protection systems for survival. Cav-1, as a key membrane-associated protein hub guiding signal transduction and nutrient delivery, may be a first-line response to chemotherapy. Studies indicate an elevation of Cav-1 in cancer cells after chemotherapeutic exposure [47]. Meanwhile, exogenous elevation or knockdown of Cav-1 expression confirmed a critical role for Cav-1 in mediating cancer chemosensitivity [9]. Other than this drug-induced response, Cav-1 may be active after radiotherapy, fluid shear stress, oxidative stress, high osmolarity, and ultraviolet exposure [139, 140]. Therefore, Cav-1 may be a stress-related molecule. Studies indicate that Cav-1 is closely associated with stress signals such as AMPK. Cav-1 silencing could induce autophagy in an AMPK-dependent pathway. Additionally, the AMPK activator metformin required Cav-1 to induce AMPK phosphorylation and an AMP/ATP ratio increase in non-small-cell lung cancer [78, 141]. Notably, Cav-1-null mice had lower body temperatures after fasting or fasting/cold treatment and this was accompanied by a significant reduction of AMPK expression [142]. Moreover, the lifespan of Cav-1-null mice was reduced, indicating that a stress-response for these mice may be weaker after Cav-1 knockout [143]. Therefore, Cav-1 might be a key stress-related molecule protecting cells from hazardous stimulus. This explains Cav-1's behavior in cancer development. During cancer initiation, malignant transformation may be accelerated due to Cav-1 loss and this would sensitize normal cells to oncogenic events. In contrast, when cancer progress to later stage and is treated, expression of Cav-1 would be upregulated to protect cancer cells escape death by speeding aerobic glycolysis, increasing stem cell populations or overexpressing ABC transporters (Figure 3) [46,144,145,146]. Nevertheless, this model may be only restricted to Cav-1 enriched normal cells such as mammary and lung epithelial cells. Since not all normal cells express Cav-1 and not all cells with low/no Cav-1 expression are primary cancer cells, Cav-1 might be just one of critical molecules responsible for assisting cells defending harmful stress. Beside Cav-1, a number of molecules including glucose regulated proteins (GRP78, GRP94, GRP170) [147], some miRNAs (miRNA-25, miRNA-30c, miRNA-23a, miRNA-200b, etc) [148] or LncRNAs (UCA1, H19, MRUL, etc) [149], were also recorded as stress-response targets. Therefore, the value of Cav-1 as a diagnostic or therapeutic target to improve chemoresistance is still needed to be validated on independent cancer type. On the other hand, molecular networks underlying Cav-1 and other stress signals must be understood by using conditional knockout models and xenografts to study its precise role in carcinogenesis or chemosensitization process in the future.

Taken together, an improved understanding of Cav-1 regulation and its role in chemosensitivity modulation is required to improve cancer prevention and therapy. Drugs targeting Cav-1 are expected to be developed and thereby provide powerful tools to explore Cav-1 molecular biology. Furthermore, larger clinical studies are needed to confirm the pathological significance of Cav-1 in cancer onset and response to conventional therapy.

## Abbreviations

MDR: multidrug resistance
abc: atp binding cassette
mapk: mitogen-activated protein kinase
cscs: cancer stem cells
cav-1: caveolin-1
csd: cav-1 scaffolding domain
egf: epidermal growth factor
pdgf: platelet-derived growth factor
pi3k: phosphatidylinositol 3-kinase
pdk1: 3-phosphoinositide-dependent protein kinase-1
dlc-1: deleted in liver cancer 1
nsclc: non-small cell lung cancer
aacr: American association for cancer research
aldh1: aldehyde dehydrogenase 1
mscs: mesenchymal stem cells
fdg-pet: fluorodeoxyglucose positron emission tomography
glut: glucose transporter
mct: monocarboxylate transporter
ampk: amp-activated protein kinase;
